# Uncertainty in multispectral lidar signals caused by incidence angle effects

**DOI:** 10.1098/rsfs.2017.0033

**Published:** 2018-02-16

**Authors:** Sanna Kaasalainen, Markku Åkerblom, Olli Nevalainen, Teemu Hakala, Mikko Kaasalainen

**Affiliations:** 1 Finnish Geospatial Research Institute Institute – FGI, Department of Navigation and Positioning, Geodeetinrinne 2, 02431 Masala, Finland; 2 Tampere University of Technology, Laboratory of Mathematics, 33101 Tampere, Finland; 3 FGI, Department of Remote Sensing and Photogrammetry, Geodeetinrinne 2, 02431 Masala, Finland

**Keywords:** hyperspectral, laser scanning, vegetation, incidence angle

## Abstract

Multispectral terrestrial laser scanning (TLS) is an emerging technology. Several manufacturers already offer commercial dual or three wavelength airborne laser scanners, while multispectral TLS is still carried out mainly with research instruments. Many of these research efforts have focused on the study of vegetation. The aim of this paper is to study the uncertainty of the measurement of spectral indices of vegetation with multispectral lidar. Using two spectral indices as examples, we find that the uncertainty is due to systematic errors caused by the wavelength dependency of laser incidence angle effects. This finding is empirical, and the error cannot be removed by modelling or instrument modification. The discovery and study of these effects has been enabled by hyperspectral and multispectral TLS, and it has become a subject of active research within the past few years. We summarize the most recent studies on multi-wavelength incidence angle effects and present new results on the effect of specular reflection from the leaf surface, and the surface structure, which have been suggested to play a key role. We also discuss the consequences to the measurement of spectral indices with multispectral TLS, and a possible correction scheme using a synthetic laser footprint.

## 1. Introduction

Multispectral terrestrial laser scanning (TLS) enables the study of target identification and analysis from their physical and biochemical properties in three dimensions. This is carried out by using the spectral indices retrieved for each point in the laser scanner point cloud from calibrated intensities of the laser returns [1–3]. The recent advances in multispectral laser scanning and its applications in different fields of remote sensing, including the most recent applications to vegetation, have been extensively reviewed in [4,5]. While the scope of this paper is in the wavelength dependency of lidar incidence angle effects and their consequences in the measurement of vegetation spectral indices, we provide in this section a summary on what has so far been observed on leaf angle effects on laser backscatter intensity from leaf surfaces.

Vegetation spectral indices are widely studied in passive optical reflectance spectroscopy to monitor, e.g. leaf pigments and other crucial vegetation properties, as well as to model leaf optical properties (e.g. [6,7] and references therein). These properties are related to vegetation status and environmental conditions in general. This information is important in understanding the dynamics of climate change and the global carbon cycle [1,3]. The angular dependence of spectral indices on wavelength is not yet known in enough detail to be able to calibrate the spectral indices measured with multi-wavelength terrestrial laser scanning. This is partially because the role of measurement geometry has only become more important with the introduction of multi-wavelength lidars to the vegetation spectroscopy scheme. With passive remote sensing, the measurement usually spans a larger area and the leaf angle effects are averaged. The capability of TLS to capture the surface properties in three dimensions enables one-shot intensity and structural information, e.g. for identifying dry parts in tree canopy [3]. The advance this facilitates for ecological studies [4] is likely to drive the development of new instruments, including multi-wavelength ones, in the near future.

Incidence angle effect on lidar backscatter intensity has been studied for about a decade with commercial monochromatic terrestrial laser scanners [8,9], although its role in the laser scanner intensity correction has been discussed much earlier (see [10] and references therein). The possible wavelength effects of incidence angle were recently discussed by [3] in the case of multispectral laser scanning. It was suggested that the differences in incidence angles were similar at different near-infrared (NIR) wavelengths. Leaves have been assumed to be Lambertian scatterers (see [11] and references therein), but some of the recent findings have suggested that in some cases, specular reflections might complicate the incidence angle behaviour at different wavelengths, and hence the measurements of spectral indices at different leaf angles [11–14]. Table 1 presents a summary of the studies on leaf (incidence) angle effects at different laser wavelengths and sampling schemes. The results have varied for different types of leaves.

**Table 1.** Summary of leaf angle effects at different laser wavelengths.

wavelengths	leaves	results	ref.
532 and 658 nm (Green Economic Chlorophyll Observation GECO)	Winter wheat (*Triticum aestivum*) and Tobacco plant (*Nicotiana benthamiana*)	Specular reflection observed for both leaves. Stronger in red for the (shinier) wheat leaf.	[11]
556, 670, 700 and 780 nm (multi-wavelength canopy lidar MWCL)	Oriental plane (*Platanus orientalis*)	No signs of specular reflection.	[15]
785 nm (FARO LS880)	Conference pear (*Pyrus Commmunis*)	No signs of specular reflection.	[16]
555, 624, 691, 726, 760, 795, 899 and 1000 nm (the FGI HSL)	Chinese hibiscus (*Hibiscus rosa-sinensis*), Zanzibar Gem (*Zamioculcas zamiifolia*), Rose (*Rosa* spp.)	Specular reflections at visible wavelengths caused differences in vegetation indices.	[13]
690 nm and 1550 nm (Leica HDS6100 and FARO X330)	Small-leaved Lime (*Tilia cordata* L.), Silver birch (*Betula pendula* L.), Norway maple (*Acer platanoides* L.), Norway spruce (*Picea abies* L.) and Scots pine (*Pinus sylvestris* L.)	Correction of specular backscatter did not improve equivalent water thickness estimation. (The angular range was small.)	[14]
1545 nm and 1063 nm (Salford Advanced Laser Canopy Analyser SALCA)	Eucalyptus (species unknown), Lily (*Spathiphyllum*), and Laurel (*Laurus nobilis*)	Greater effect at 1063 nm than at 1545 nm, still negligible for normalized difference index. Specular peak for dry eucalyptus.	[5]
1550 nm (RIEGL VZ-400)	Piggyback Plant (*Tolmiea menziesii*)*,* Smooth Hydrangea (*Hydrangea arborescens*)*,* Rhododendron (*Rhododendron* sp.)*,* Garden Croton (*Codiaeum variegatum*)*,* Red Robin (*Photinia fraseri*)*,* Dwarf Umbrella Tree (*Schefflera arboricola*)*,* Ficus Tree (*Ficus benjamina*) and Zanzibar Gem (*Zamioculcas zamiifolia*)	Strong specular reflection for shiny leaves, high diffuse fraction for rough leaves.	[12]

In their analysis of crop foliar nitrogen with a dual wavelength laser system (green and red), [11] observed effects of leaf angle variation, which were explained to be related to specular reflections, especially from shiny leaf surfaces and near 680 nm, i.e. the chlorophyll absorption maximum. They suggested that choosing laser wavelengths with more similar leaf bidirectional reflectance distribution functions and having a higher isotropic reflectance component might be able to reduce the leaf angle effect. A strong specular backscatter at zero (perpendicular) incidence was also observed in [12] at 1550 nm for eight broadleaf samples, their surface properties varying from shiny to hairy matte. Strong specular reflection was found for the shiniest species, whereas the specular fraction was lower for the most matte ones. The results also suggested that rough leaves might have higher diffuse fractions. Overall, the results suggest that the backscattering specular reflection is related to leaf surface structure. Our previous study with the hyperspectral lidar [13] showed similar results to [11], i.e. strong specular reflections near the normal incidence at visible wavelengths. Summarizing all the results so far (table 1), it appears that the specular effect (and hence the difference in the incidence angle behaviour) is different at different wavelengths, especially between visible and NIR, and is greater for waxy or shiny leaves in the visible region. For rough and matte leaves the specular effects were smaller or completely negligible, and the difference between visible and NIR was not so substantial.

The aim of this paper is to quantify the wavelength effects on the incidence angle behaviour for different leaf surfaces and to discuss their consequences in the retrieval of vegetation spectral indices with multispectral lidar. In our previous study [13] we studied the role of specular reflections and found them to be stronger in visible for waxed leaves. To study further the role of leaf surface roughness, we present results for a larger number of samples, also including non-waxy deciduous leaves as well as conifer needles and shoots. We use a simple model to get some idea on the possible role of leaf properties in the incidence angle behaviour. Even if the leaf surface structure explained the incidence angle effects, it would not provide a means for removing them. Instead, our study provides an estimate of the resulting measurement errors and how they should be taken into account in the measurements.

## 2. Material and methods

**Figure 1.**
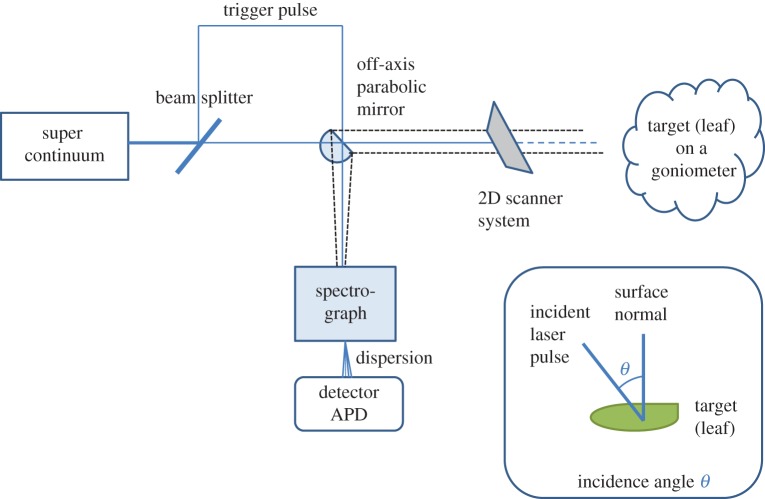
The measurement set-up of the hyperspectral lidar (HSL). (Online version in colour.)

The lidar measurements of leaves and shoots were carried out with the Finnish Geospatial Research Institute Hyperspectral Lidar (FGI HSL) [2], which is an eight-channel full waveform digitizing laser scanner prototype based on supercontinuum laser technology (figure 1 and table 2 for more details). The operation principle is the same as in a monochromatic pulse-based terrestrial lidar: the range measurement is based on the time-of-flight of the returned laser pulse. The output point cloud (*x,y,z,I*) contains the intensity *I* as a function of wavelength, in this case, an eight-channel spectrum (500–1000 nm) is associated with each point (*x,y,z*). As only eight channels are currently digitized, the detector system is multispectral, but the wavelength channels can be selected by adjusting the spectrograph position, i.e. the dispersion, with respect to the avalanche photodiode array to detect different wavelengths. This also explains the slight changes in the centre wavelengths of different channels between measurements. In our calibration studies, we have observed a 6% approximate error level (a standard deviation of the peak height measurement) in the reflectance measurement of the HSL detector. The error is consistent for all wavelengths at the range measured.

**Table 2.** The FGI HSL instrument specifications. See also [13]. More details on the channel selection are available in [2].

centre wavelengths of channels (1–8)	564.3, 610.8, 659.9, 720.3, 764.8, 818.0, 878.6 and 979.2 nm
optical bandpass	20 nm Full Width at Half Maximum (FWHM)
pulse rate	5.3 kHz
pulse length	1 ns
average output power	41 mW (LEUKOS-SM)
beam diameter	4 mm at exit, 5 mm at 4 m for 543 nm
beam divergence	∼0.02° at 543 nm
range resolution	15 cm
scan speed	Max 60°/s (vertical)

The leaf and needle samples measured were leaf samples from Silver birch (*Betula pendula*) and Norway maple (*Acer platanoides*), a shoot sample from Norway spruce (*Picea abies L.*) and needles and a shoot from Scots pine (*Pinus sylvestris L*.), for which the needles were sampled by attaching them side by side on a fabric, with either adaxial or abaxial sides facing the scanner. The fabric was black with a low reflectance, to minimize the effects of partial laser returns from the fabric through some gaps between the needles. The measurements were carried out in laboratory conditions. The samples were placed on a motorized rotating platform at about 4 m distance from the scanner. The beam diameter at 4 m distance is 5 mm at 543 nm (table 1). The beam size calculation is based on the figures provided by the laser manufacturer, but the beam divergence is known to increase with increasing wavelength. This will affect the spot size at near-infrared wavelengths. To reduce the effect of spot size on the intensity, the distance calibration is done separately for each channel.

The incidence angle was changed in 5° increments, and a two-dimensional scan over the sample was performed to produce a point cloud at each incidence angle. The resulting HSL point clouds were processed using Matlab 2013a software (The MathWorks^®^, Inc). Laser echoes from outside the leaf or needle sample were manually cropped from the point clouds. The point spacing could be manually specified for each measurement, but a typical sampling was about 7 vertical and 20 horizontal points per cm, resulting in a few hundred points (averaged from 10 pulses) per sample. The mean intensity of all the echoes from the sample was used to calculate the backscattered reflectance at each incidence angle. The intensity calibration was carried out with a 99% Spectralon^®^ reference target, scanned at the same distance as the targets. We also included the samples measured in [13] into the analysis. Those were Chinese hibiscus (*Hibiscus rosa-sinensis*), Zamioculcas (common name ‘Zanzibar Gem’) (*Zamioculcas zamiifolia*) and a Rose (*Rosa* spp.) commonly available in florist shops (see also table 1).

An analysis similar to [12] was carried out to study whether the specular component is wavelength dependent and to explore the relationship between surface roughness and the specular reflection. A linear combination of Lambertian law and the Beckmann model, which introduces a specular component [12], is also related to the surface roughness of the target:2.1
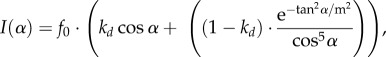
where *I*(*α*) is the backscatter intensity at incidence angle *α*, *f*_0_ is the intensity at normal incidence angle, *k*_d_ is the fraction of the diffuse component, and *m* is the surface roughness. The values of the *k*_d_ and *m* parameters are between 0 and 1. In [12], *m* = 0 would represent a smooth surface, whereas values near 0.6 would indicate a rough surface.

Some of the samples did not follow the model of equation (2.1) even approximately. Therefore, we also fitted a second-order Fourier series to the observed *I*. This serves as an interpolating approximation for the data only, without any physical modelling. The interpolated function *I* helps in assessing the vegetation index as a smooth function of the incidence angle so that its variation reported below is not greatly affected by noise or outliers.

## 3. Results and discussion

**Figure 2.**
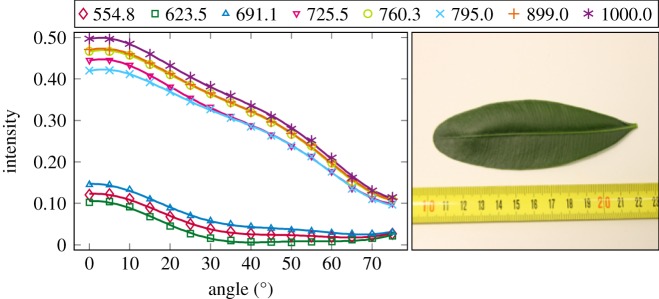
The plotted incidence angle versus laser backscatter intensity for the Zanzibar gem sample (Z Gem). The second-order Fourier series approximation fitted to the data is also shown for all wavelengths. (Online version in colour.)

**Figure 3.**
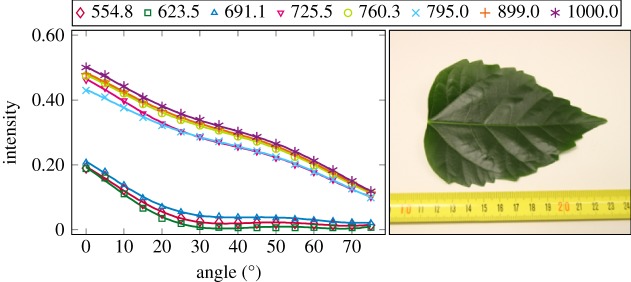
The plotted incidence angle versus laser backscatter intensity for the Chinese hibiscus sample (China rose). The second-order Fourier series approximation fitted to the data is also shown for all wavelengths. (Online version in colour.)

**Figure 4.**
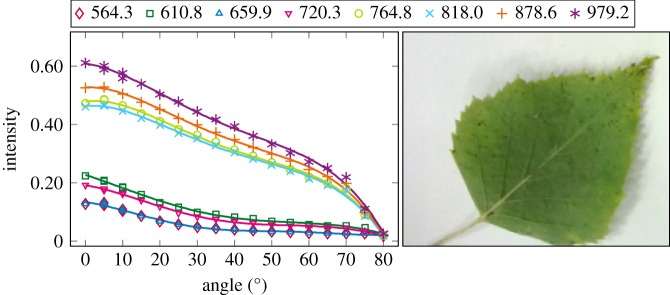
The plotted incidence angle versus laser backscatter intensity for the birch leaf. The second-order Fourier series approximation fitted to the data is also shown for all wavelengths. (Online version in colour.)

**Figure 5.**
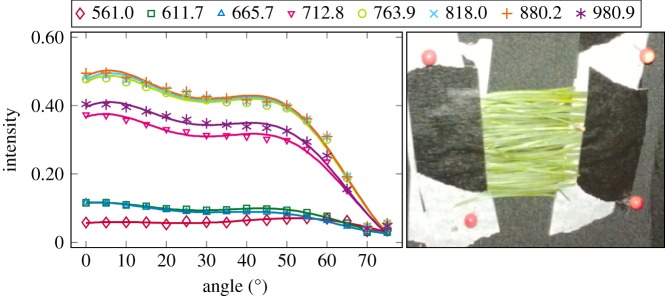
The plotted incidence angle versus laser backscatter intensity for the pine needles, abaxial side. The second-order Fourier series approximation fitted to the data is also shown for all wavelengths. (Online version in colour.)

**Figure 6.**
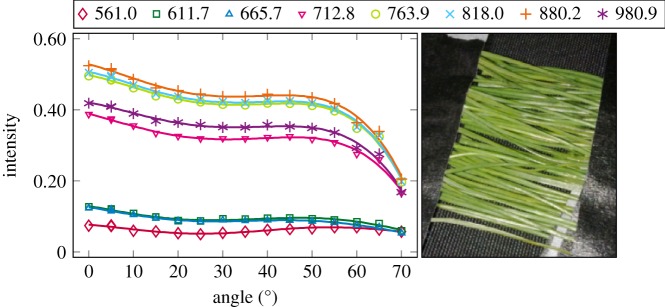
The plotted incidence angle versus laser backscatter intensity for the pine needles, adaxial side. The second-order Fourier series approximation fitted to the data is also shown for all wavelengths. (Online version in colour.)

**Figure 7.**
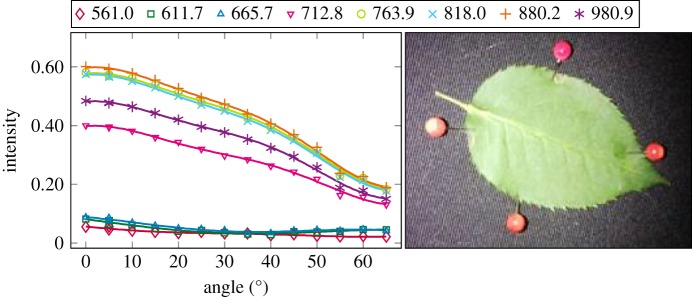
The plotted incidence angle versus laser backscatter intensity for the rose leaf. The second-order Fourier series approximation fitted to the data is also shown for all wavelengths. (Online version in colour.)

**Figure 8.**
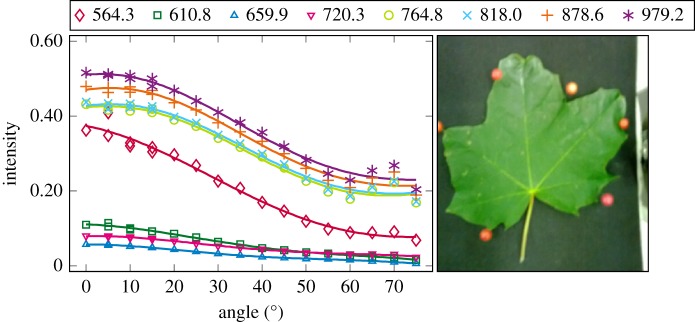
The plotted incidence angle versus laser backscatter intensity for the maple leaf. The second-order Fourier series approximation fitted to the data is also shown for all wavelengths. (Online version in colour.)

**Figure 9.**
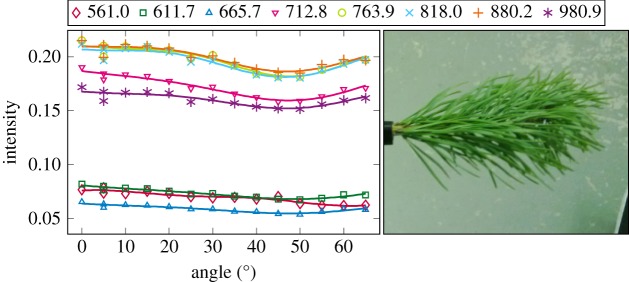
The plotted incidence angle versus laser backscatter intensity for the pine shoot measured with side towards the lidar. The second-order Fourier series approximation fitted to the data is also shown for all wavelengths. (Online version in colour.)

**Figure 10.**
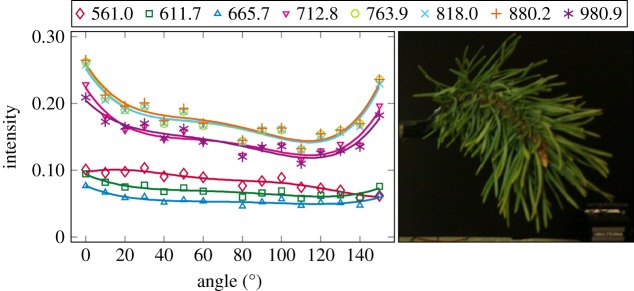
The plotted incidence angle versus laser backscatter intensity for the pine shoot measured with its top towards the lidar. The second-order Fourier series approximation fitted to the data is also shown for all wavelengths. (Online version in colour.)

**Figure 11.**
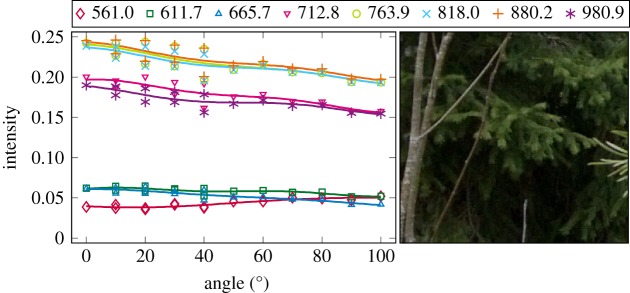
The plotted incidence angle versus laser backscatter intensity for the spruce shoot measured with side towards the lidar. The second-order Fourier series approximation fitted to the data is also shown for all wavelengths. (Online version in colour.)

The plotted incidence angle versus laser backscatter intensity curves are shown in figures 2–11. The laser backscatter is plotted as the mean of the intensity values of the points on an entire leaf or needle sample (or most of the sample) cropped from the point cloud. The whole area covered by the cropped point set thus represents the effective instrument footprint in this experiment. The standard deviations of the values of the point set varied from typically 20% to 50% in the visible (the errors being largest below 600 nm, which might result from laser and detector effects) towards 5–10% in the NIR wavelengths. Since the instrument error is about 6%, these deviations show that each point value is also dependent on the local structure of the sample (biological properties and surface features). On the other hand, the individual errors cancel out in the mean over hundreds of points as demonstrated by the smooth shapes of the curves of figures 2–11. In other words, the averaging over the effective instrument footprint (that is much larger than an individual laser spot) removes the effect of local variations at size scales smaller than the footprint but larger than the laser spot. The measurement can thus be expected to be the same for any part of a much larger sample of same target material as long as the incidence angle is kept the same. The nominal error of the intensity value of the footprint decreases rapidly as the number of laser spots in the footprint increases, regardless of the individual errors of the spot values, becoming lower than the instrument error of 6%. This statistical ‘wisdom-of-the-crowd’ phenomenon of the vanishing error of the mean of many measurements of arbitrarily large errors is described in, e.g. [17]. The averaging effect may already be a factor in the individual spots. As the laser spot size is known to increase towards NIR wavelengths, this may explain the decrease in the spot standard deviations towards larger wavelengths. Another factor is the strong absorption that results in a weaker signal in the visible than in the NIR.

**Figure 12.**
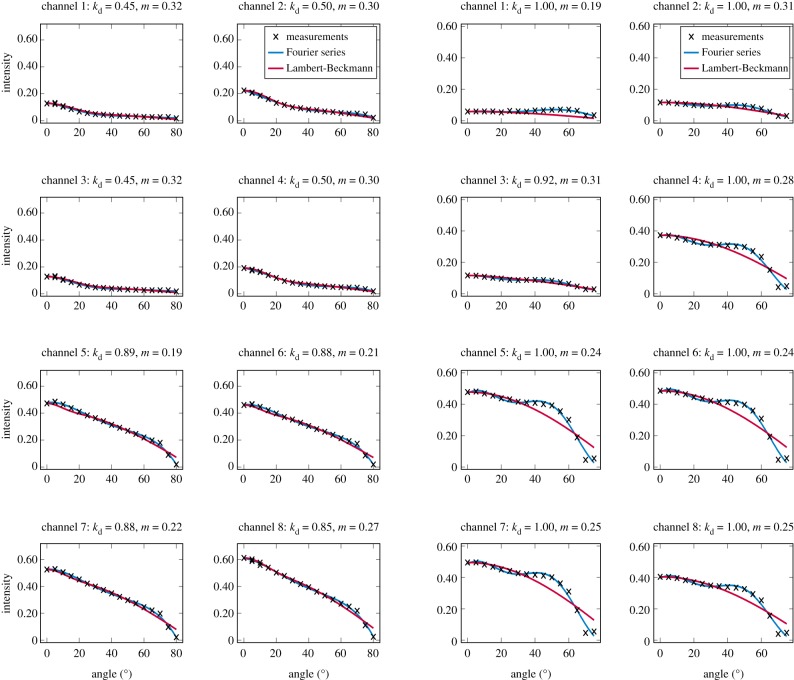
Lambert–Beckmann and Fourier fits for the birch sample (left) and the pine needles abaxial side (right). The fit is not so good at NIR wavelengths for the needle sample, which must also be taken into account in the interpretation of the parameter values in tables 3 and 4. (Online version in colour.)

The measured angle–intensity data were fitted with a Lambertian–Beckmann curve from equation (2.1). The non-linear curve fitting was performed in Matlab using the Trust Region Reflective algorithm to optimize the values of the *k*_d_ and *m* parameters, whose values were limited between 0 and 1. The fitting was performed for each channel separately. Resulting values for the diffuse fraction parameter *k*_d_ are listed in table 3 and those for the surface roughness parameter *m* in table 4. When the parameter *k*_d_ value equals one, the surface has no specular component. Additionally, the measurements were fitted with a second-order Fourier series approximation. Both fitted curves for each sample and channel are visualized in figures 2–11. For leaf samples, *k*_d_, i.e. the diffuse component, appears to grow towards NIR wavelengths, which indicates stronger specular reflections in the visible wavelength range. The surface roughness parameter *m* appears to diminish towards NIR, which is more difficult to interpret, but might indicate a different scattering behaviour at NIR laser wavelengths. For needle samples and shoots, the interpretation of *k*_d_ and *m* would not make sense as the Lambert–Beckmann function did not fit the data. Examples of the Lambert–Beckmann fits for one leaf and needle sample are given in figure 12.

**Table 3.** Values of the optimized diffusion component parameter *k*_d_ for the different samples and channels (see table 2 for wavelength channels).

sample	channel							
	1	2	3	4	5	6	7	8
ZGem	0.37	0.22	0.42	0.84	0.89	0.88	0.88	0.87
China rose	0.34	0.31	0.37	0.73	0.77	0.76	0.77	0.77
birch	0.45	0.50	0.45	0.50	0.89	0.88	0.88	0.85
pine abaxial	1.00	1.00	0.92	1.00	1.00	1.00	1.00	1.00
pine adaxial	1.00	1.00	0.89	1.00	1.00	1.00	1.00	1.00
rose	0.66	0.50	0.54	0.84	0.87	0.86	0.86	0.85
maple	0.58	0.56	0.50	0.76	0.92	0.93	0.94	0.93

**Table 4.** Values of the optimized surface roughness parameter *m* for the different samples and channels.

sample	channel							
	1	2	3	4	5	6	7	8
ZGem	0.29	0.34	0.29	0.20	0.19	0.18	0.19	0.19
China rose	0.38	0.40	0.37	0.33	0.30	0.31	0.30	0.31
birch	0.32	0.30	0.32	0.30	0.19	0.21	0.22	0.27
pine abaxial	0.19	0.31	0.31	0.28	0.24	0.24	0.25	0.25
pine adaxial	0.44	0.44	0.37	0.38	0.32	0.32	0.32	0.32
rose	0.33	0.30	0.32	0.22	0.18	0.19	0.19	0.19
maple	0.27	0.27	0.30	0.22	0.18	0.18	0.18	0.19

In some cases such as in figure 10, the angle dependence of the curve is not monotonic as one would expect physically. This is probably caused by systematic effects in the measurement such as the more pronounced effect of the stochastic geometry of the shoot at high angles. The targets with more ordered surfaces show essentially monotonous dependence.

As an example of variation in spectral vegetation indices, table 5 lists the normalized difference vegetation index (NDVI) and water index (WI) values for each sample. The NDVI is calculated using reflectance values (*R*) at two wavelengths in NIR and visible, i.e. both sides of the spectral red edge (e.g. [1]). In this study, we used 691 nm in the red and 795 nm in NIR for Zanzibar Gem and China rose, and 666 and 818 nm for pine and spruce samples:3.1
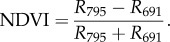
We also compared the water concentration index (WI) used in, e.g., [18]:3.2
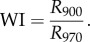

**Table 5.** Minimum and maximum values of the NDVI and WI for each sample.

sample	NDVI min	NDVI max	WI min	WI max
Z Gem	0,49	0,74	0,95	0,96
China rose	0,35	0,74	0,96	0,98
birch	0,50	0,78	0,86	0,91
pine abaxial	0,22	0,66	1,15	1,23
pine adaxial	0,58	0,67	1,22	1,26
rose	0,62	0,85	1,24	1,27
maple	0,76	0,93	0,90	0,96
P shoot side	0,53	0,54	1,22	1,27
P shoot top	0,47	0,57	1,18	1,29
S shoot	0,58	0,65	1,27	1,32

As 900 and 970 nm were not available in this measurement, we used the wavelengths closest to these values for each sample. Table 5 shows large variations in the spectral indices, some of which, however, may result from, e.g., the intensity dropping fast towards large angle of incidence (as for the Birch NDVI 0.05 at 80°, also cf. figure 12). However, this will also be the case in field experiments for entire trees, as the laser beam hits the tree parts at all possible angles.

The sample results for NDVI and WI show that the vegetation indices change with the laser incidence angle. If these indices change, then any wavelength-dependent index will. We used the Lambert–Beckmann model to obtain clues for the incidence angle effect rather than to model it accurately in a quantitative sense. We discovered some systematic, monotonic trends. As the error is geometric, and not instrumental, it cannot be corrected with any modelling, because the leaf angle is usually difficult to retrieve in the measurement of large targets, such as tree canopies.

## 4. Conclusion

The main focus of this paper was to study the measurement of vegetation spectral indices with multi-wavelength terrestrial lidars, and provide a practical assessment on how the leaf surface material and structure affects the incidence angle behaviour. The main result of our study is that there is a previously unknown systematic error, which has to be taken into account. This is not an instrumental error but results from changes in the incidence angle. The objective of our study was to find and report a lower limit to this error. Even if we were able to model the signal and leaf behaviour perfectly, it is not enough to correct for the incidence angle effect as we do not know the incidence angle.

The results show that in many cases, typically with waxy leaves (target surfaces apparently smooth under the footprint), the quantitative change in a vegetation index is several tens of per cent between the broadside-on (0°) and edge-on (90°) orientations. Therefore, the leaf-orientation effect plays a significant part in the interpretation of measurements. The Lambert–Beckmann model appears to offer a consistent explanation for the angle effect in the waxy leaf case. The different wavelengths ‘see’ the leaf structure differently; for instance, the material appears to be more specular for visible wavelengths. In this case, either the incidence angle effect should be corrected, or, if the leaf angle is not known, its effect on the results (e.g. retrieval of tree properties such as water content from spectral indices) must be quantified as a systematic error. This error would result in noise of tens of per cent between nearby sample points in a tree. The error is inevitable regardless of the accuracy of the data and cannot be corrected with any physical modelling for an individual point.

On the other hand, for targets that are rougher (stochastic) under the footprint of the instrument, such as the pine shoot, the index variation appears to be small. Therefore, the key issue is the averaging of the geometric effects over the laser footprint large enough for the geometry to be stochastic at the footprint scale. This suggests error reduction by a synthetic laser footprint, i.e. the average value of several nearby samples that includes the stochastic structure in the same way that the pine shoot already does for the footprint used in this experiment. For leaves, this means averaging over a number of nearby leaves at various incidence angles. Naturally this decreases the spatial resolution somewhat, but it should essentially remove the angle-dependence error when the sampling size of the synthetic footprint is large enough. In any case, the index value from a single laser spot is likely to have a large essentially random error and averaging over several spots is necessary in the first place as discussed above.

As an example of a large footprint, strong correlation was found in [19] between foliar nitrogen concentration and averaged laser return intensity at 532 nm for wheat leaves. In a future paper, we plan to use a leaf-augmented quantitative structure model ([20] and Åkerblom [21]) to model the stochasticity of the leaf orientation. This helps to quantify the systematic effects between various parts of the tree (for example, potential ‘limb darkening’ effects as the central parts of the tree, as seen from the instrument, may contain more broadside-oriented leaves than the limb parts). We can also determine the resolution scales in which the spectral indices are measurable as the best compromise between systematic errors and spatial resolution.

## Data accessibility

The datasets supporting this article have been uploaded to http://math.tut.fi/inversegroup/datasets/kaasalainen2017uncertainty.
